# Beyond the Biomedical: Community Resources for Mental Health Care in Rural Ethiopia

**DOI:** 10.1371/journal.pone.0126666

**Published:** 2015-05-11

**Authors:** Medhin Selamu, Laura Asher, Charlotte Hanlon, Girmay Medhin, Maji Hailemariam, Vikram Patel, Graham Thornicroft, Abebaw Fekadu

**Affiliations:** 1 Department of Psychiatry, College of Health Sciences, School of Medicine, Addis Ababa University, Addis Ababa, Ethiopia; 2 Centre for Global Mental Health, London School of Hygiene and Tropical Medicine, London, United Kingdom; 3 Centre for Global Mental Health, Institute of Psychiatry, King’s College London, London, United Kingdom; 4 Aklilu Lemma Institute of Pathobiology, Addis Ababa University, Addis Ababa, Ethiopia; 5 Centre for Affective Disorders and Affective Disorders Research Group, Department of Psychological Medicine, Institute of Psychiatry, King’s College London, London, United Kingdom; Hangzhou Normal University, CHINA

## Abstract

**Background:**

The focus of discussion in addressing the treatment gap is often on biomedical services. However, community resources can benefit health service scale-up in resource-constrained settings. These assets can be captured systematically through resource mapping, a method used in social action research. Resource mapping can be informative in developing complex mental health interventions, particularly in settings with limited formal mental health resources.

**Method:**

We employed resource mapping within the Programme for Improving Mental Health Care (PRIME), to systematically gather information on community assets that can support integration of mental healthcare into primary care in rural Ethiopia. A semi-structured instrument was administered to key informants. Community resources were identified for all 58 sub-districts of the study district. The potential utility of these resources for the provision of mental healthcare in the district was considered.

**Results:**

The district is rich in community resources: There are over 150 traditional healers, 164 churches and mosques, and 401 religious groups. There were on average 5 *eddir* groups (traditional funeral associations) per sub-district. Social associations and 51 micro-finance institutions were also identified. On average, two traditional bars were found in each sub-district. The eight health centres and 58 satellite clinics staffed by Health Extension Workers (HEWs) represented all the biomedical health services in the district. In addition the Health Development Army (HDA) are community volunteers who support health promotion and prevention activities.

**Discussion:**

The plan for mental healthcare integration in this district was informed by the resource mapping. Community and religious leaders, HEWs, and HDA may have roles in awareness-raising, detection and referral of people with mental illness, improving access to medical care, supporting treatment adherence, and protecting human rights. The diversity of community structures will be used to support rehabilitation and social reintegration. Alcohol use was identified as a target disorder for community-level intervention.

## Background

Mental disorders cause substantial personal and societal burden worldwide. Low and middle-income countries (LMICs) bear most of the burden [1]. Emphasis is typically placed on the large treatment gap in these settings, indicating the percentage of individuals who require care but do not receive formal treatment. For example, in rural Ethiopia, the treatment gap for people with schizophrenia is 90% [2]. The scarcity of specialist human and material resources, low policy prioritisation and low levels of community awareness are considered to be key reasons for the widespread lack of access to formal care [3,4]. In most sub-Saharan African countries, including Ethiopia, the number of mental health professionals is extremely low and mental health care is provided by centralised institutions located in capital cities which are inaccessible to the majority [5]. As a consequence, people with mental illness in Ethiopia usually rely on family support, traditional healers and holy water sites for their care [6]. Holy water, accessed at specific sites associated with the Orthodox Church, is believed to have curative properties. Lack of access to formal treatment manifests in high levels of disability [7] and mortality [8] amongst people with mental illness and a heavy burden on caregivers [9,10]. Physical restraint at home and at holy water sites is relatively common amongst people with schizophrenia in Ethiopia.

The use of existing formal health care structures, including non-specialist health workers in the form of task sharing, is widely advocated for the scale up of mental health interventions in settings with limited resources [3,11–15]. For example, the World Health Organisation (WHO) Mental Health Gap Action Programme (mhGAP) advocates that mental health should be integrated into primary care in order to address the unmet mental health needs in low and middle income countries [16]. The 2012 Ethiopian Mental Health Strategy aligns with the mhGAP, focusing on the expansion of mental healthcare across the country through integration into primary care [5].

However, the development of integrated mental health care tends to focus solely on clinical needs and gaps without attention being paid to local resources and strengths [16]. For example, WHO estimations of required resources for scaling up usually focus on costs related to biomedical services. This top down model does not consider the potential role of local customs, norms and resources and leaves little room for community participation. Furthermore, health facility-based mental healthcare in isolation will be unsustainable and unable to meet the broader social needs of people with mental illness, such as dealing with poverty and human rights [17]. A person’s community can have a powerful influence, both positive and negative, on their mental illness, including their capacity to access and utilise medical care, their experiences of stigma, their recovery and reintegration. This is particularly the case in settings such as rural Ethiopia, where mental health literacy is low [6] and the vast majority of individuals with mental illness are cared for at home [2,6].

The focus of discussion in addressing the treatment gap is often on what biomedical services can offer. A broader and more pluralistic approach is required. Existing community resources can be capitalised upon in order to enhance mental health care delivered at the primary care and community level [13,18]. In this context, community resources or assets may be features of individuals, organizations, or the built environment that relate to health [19]. They can be either formal or informal structures [18,20].

There are several examples of mental health interventions utilising existing community resources to maximise their impact. Integrating mental health interventions into existing non-governmental organisations (NGOs), in particular community-based rehabilitation (CBR) programmes, has been identified as a way to overcome problems with lack of capacity and resources [13,17,21–24]. In Nepal, Ghana and India, people with mental illness have been incorporated into existing self-help groups and micro-credit schemes run by local NGOs [13,22,24].

Community leaders and traditional providers may also represent major resources that can be leveraged for the benefit of patients, though there has been little research into how this type of engagement can be put into practice [3]. Working in collaboration with traditional healers can improve the acceptability of mental health services [6,23,25]. In Bali, local spiritual healing practices were successfully integrated with biomedical services to reduce physical restraint amongst people with severe mental illness [26]. Community leaders, including parish priests and village chiefs, have facilitated access to mental healthcare as part of CBR and community mental health projects in India and Nigeria [21,27].

Resource mapping enables a systematic inventory of community resources that have the potential to be mobilised and used in the development of a scalable health service with community buy-in, particularly in low-income countries. Resources may include services, support mechanisms, information, social capital and infrastructure available and accessible within a specified community. The focus is on recognising strengths and opportunities as opposed to deficiencies and needs [28,29]. According to McKnight and Kretzmann resource mapping [29] “The process of identifying capacities and assets, both individual and organizational, is the first step on the path toward community regeneration”. Resource mapping allows active participation of the community in the development of health services. This is relevant in settings with few material resources where, despite improvement in services, the community is likely to carry a substantial proportion of the care burden. Engaging the community in mental health care is therefore one of the best ways to ensure holistic care, the continuity of care [30] and potentially better outcomes for individuals. Yet most of the peer-reviewed literature on resource mapping comes from high-income countries [19,28,31,32] and there are no studies relating to mental health interventions to our knowledge. Furthermore there is little explicit evidence for the influence of resource mapping on the design of health interventions. As part of a project to develop an integrated mental health service in rural Ethiopia, the Programme for Improving Mental health care (PRIME), we mapped available community resources in order to use these resources in the implementation of integrated care. Once the resources were mapped we intended to mobilise the community assets to enhance mental health literacy, community based rehabilitation and community based supports. In addition, through the resource mapping process, PRIME was interested to identify potential risk factors that may be addressed, by utilisation of community resource or direct intervention of the project.

## Aim

To map community resources available in Sodo district, Ethiopia, and identify environmental risk factors in order to guide development of a relevant, comprehensive and effective mental health care plan (MHCP).

## Method

### Ethics statement

This study received ethical approval from the College of Health Sciences Institutional Review Board, Addis Ababa University (084/11/PSY) as part of a baseline situational appraisal. Data collected was purely related to community assets or resources and no personal information was included. No information with potential negative impact on the community was collected. The study was conducted with the full agreement of the district. Because of this only oral consent was obtained from the key informants. This is consistent with other studies of asset mapping. However, participation in the study as key informant was voluntary.

### Setting

The study was conducted in Sodo district, Gurage zone, located in the Southern Nations, Nationalities and People’s Regional state of Ethiopia. Bui, the capital of the Sodo district is located about 100 km south of Addis Ababa, the capital of Ethiopia. Sodo district covers a geographic area of 830. 63 km^2^ and comprises 54 rural and 4 urban sub-districts (smallest administrative units). Most people live in one-roomed mud and straw houses and work as subsistence farmers. Only 21.5% of people are literate. The majority is orthodox Christian and the ethnic composition of the population is Gurage 85.3%, Oromo 11.6% and Amhara 1.5%. Malaria and cutaneous leishmaniasis are important endemic health problems of the district [33].

### The PRogramme for Improving Mental healthcare (PRIME)

The current study is part of a wider study, the PRogramme for Improving Mental healthcare (PRIME) project, which aims to provide evidence to support the implementation and scale up of mental health care in primary care in five low and middle-income countries (Ethiopia, India, Nepal, South Africa and Uganda) [34]. The initial stage was to develop a comprehensive MHCP for Sodo district for selected priority mental and neurological problems: psychosis, depression, alcohol use, maternal mental disorders and epilepsy. The MHCP will later be implemented, evaluated and scaled up. The MHCP incorporates interventions at the three levels of the health system: community, health facility and health service organisation levels. A situational analysis was previously conducted to describe existing primary and community healthcare and specialist mental health services in Sodo [33]. It also attempted to describe the community environment, but because it relied on information available in the public sector, a full picture was not obtained. Moreover, the situational analysis did not attempt to map resources that could be engaged in the MHCP. To complement the situational analysis, in particular to develop the community level aspects of the MHCP, in the current study resource mapping was utilised to identify community resources. This allowed us to examine resources outside of the public sector, and also to understand the geographical spread of resources.

### Study design and assessments

The study design was a community-level cross-sectional survey. Quantitative data were collected using a community resource inventory, adapted from a hand book of community assessment developed in Canada [35], which is being used as a resource for developing community asset mapping templates in the School of Social Work of Addis Ababa University. Three 90 minute research team meetings were held to select the items relevant for the rural Ethiopian context and to exclude those that were not considered relevant (for example restaurants). Additional items that should be added, for example *eddir* groups, were also considered in the meetings. The original function of *eddir* was as a funeral association i.e. providing financial, practical and emotional support when a member dies. In the final version there were seven sections, excluding the section on general information, and 83 items, which assess various domains of community resources, including physical assets (for example forests), community associations, health facilities, education facilities, justice system, recreational venues, agriculture, religious institutions, and NGOs (See S1 File). The inventory includes open questions (for example ‘What is the main means of subsistence in this sub-district?’) and closed questions (for example ‘How many churches are there in this sub-district?’). The inventory was initially developed in English and translated into the local language, Amharic, for ease of administration by the interviewers. The inventory was back translated into English to check for conceptual equivalence.

Health Extension Workers (HEWs), who are frontline community health workers, were trained for two days in data collection. The training covered what community assets are, the purpose of identifying them and how to identify them. Two HEWs were assigned to the sub-district where they usually work, with each one covering a pre-specified geographical catchment area.

On average each HEW interviewed two key informants (people believed to have rich knowledge about the local community) to complete the inventory. In total 165 key informants were interviewed including local community leaders, sub-district chairpersons, district officials, teachers, and community elders. HEWs were collecting data in their usual place of work and residence; therefore they had good prior knowledge about appropriate key informants. Each HEW collated the information from the individual interviews to produce one complete inventory. Two inventories were therefore produced for each sub-district (one for each catchment area). The HEWs were also considered key informants; where they were unable to gather the information from community leaders, they were encouraged to fill in the outstanding information themselves.

### Data management and analysis

Prior to the analysis, the two inventories were combined to produce a sub-district level summary of community resources. As the HEWs covered distinct geographical catchment areas, overlap between the inventories was perceived to be unlikely. However the data was checked to ensure to ensure no resources were counted twice. The study investigators triangulated the information with district-level officials and community representatives.

A simple descriptive analysis was used to summarise the data. The frequency and distribution of the different resources across the district was described. The data entry was completed using EpiData Version 3.1 then exported to Stata version 11 [36] for the analysis.

## Results

The community resources identified are summarised in Table 1.

**Table 1:** Community resources in Sodo district.

Community Resources	Number	Min/Sub-district	Max/Sub-district
**Traditional healers**			
*Tanquay* (‘witch doctor’)	21	0	2
Herbalists	49	0	8
Traditional Birth Attendants	52	0	16
Bone Setters (Traditional physiotherapists)	56	0	8
**Religious healers / healing sites**			
Holy water sites	27	1	6
Church healers	Unknown		
**Religious institutions**			
Orthodox Churches	125	0	9
Mosques	11	0	1
Protestant (evangelical) churches	28	0	1
Other religious institutions	5	0	1
**Community-based organisations**			
*Eddirs*	301	3	23
*Mahaber* (non religious association)	245	5	12
*Tsewa* (religious association)	156	3	12
Women’s associations	67	1	2
Youth’s associations	58	1	1
**Education institutions**			
Adult literacy groups	45	0	1
Primary School	54	0	2
Secondary School	2	0	1
**Microfinance**			
Micro-finance groups	51	0	16
**Non-governmental organisations**			
Compassion (Supporting vulnerable children)	8	0	1
**Alcohol establishments**			
*Telabet* (where home-brewed beer is consumed)	50	1	19
*Tejbet* (where home-brewed honey wine is consumed)	22	0	5
*Arakebet* (where home-distilled spirits are consumed)	50	2	15
**Health facilities**			
Health centres	8	0	1
Health posts	58	1	1
**Justice system**			
Social courts	58	1	1
Police stations	2	1	0

### Public sector health resources

Health centres, staffed by nurses and health officers, and health posts (satellite clinics), staffed by HEWs, constituted the public sector biomedical resources. There are eight health centres in the district and one health post for each of the 58 sub-districts. Each health centre provides primary healthcare for 20,000–25000 people, but they do not currently cover mental health. 21 sub-districts had two HEWs in post, 32 sub-districts had one HEW and there were five sub-districts with no HEW currently in post. Each HEW is responsible for the health of 1000 households. They make house-to-house visits to deliver family planning, antenatal care, sanitation advice, malaria prevention, and give advice on the effects of harmful traditional practices. A bridge between the biomedical services and the community resources are the Health Development Army (HDA). HDAs are a network of community health volunteers established through government initiative. Of a group of five household heads, one is trained by the HEW on health promotion and prevention, for example sanitation. That household head then becomes an HDA member and trains the remaining four household heads. These household heads go on to disseminate the information to their immediate community. The aim is to create a platform for awareness-raising about communicable and non-communicable diseases and social issues. HEWs are also responsible for leading ‘Community Conversations’ groups, another bridge aimed at disseminating information on a range of health and social topics to the general population.

### Traditional and faith based practitioners

Various types of traditional healers were identified. There are 49 herbalists, 21 *tanqway* (‘witch doctors’), 52 traditional birth attendants and 56 bonesetters in Sodo district. Bonesetters use splints and massage to heal injuries and fractures. The herbalists prepare herbal preparations to treat a range of illnesses including infectious diseases such as malaria and hepatitis (*yewof beshita)* and mental and neurological disorders such as epilepsy. *Tanqway* tend to be consulted for conditions perceived to have a supernatural cause, for example infertility and psychosis. They treat using tinctures, animal sacrifices, and rituals involving the individual. In addition there are 27 holy water sites, each one of which has several holy water priests and attendants attached to it. Holy water is typically a first port of call when a family member has a condition perceived to be triggered by spiritual power, such as severe mental illness. People drink the holy water or are baptised in it to gain the benefits.

### Community-based organisations

A number of community-based organisations (CBOs) were identified. In general these had been established by community members with the aim of providing mutual support. On average there were five *eddir* groups in each sub-district. Each group has about 150–200 households as members. The original function of *eddir* was as a funeral association i.e. providing financial, practical and emotional support when a member dies. But the role of *eddir* groups also includes providing support to members experiencing various social, economical and health problems. *Eddirs* are highly organised and structured with a chairperson and secretary. The *eddir* leaders are also highly respected and their recommendations and admonitions stand. They have power to ostracize a community member or mobilise the community in support of a member.

There are 245 *mahaber* groups and 156 *tsewa* groups across the district. These non-governmental community based associations are founded and run by community members. The groups tend to have 10–20 members and meet monthly in members’ homes. *Tsewa* have an Orthodox Christian focus; activities include group prayer, singing and meals. *Mahaber* groups are similar, but may be focused around other shared interests, for example local development projects.

There are 67 women’s and 58 youth associations across the district. These government associations, which have monthly meetings, are organised by HEWs or sub-district officials. The aim of the women’s associations is to empower women through raising awareness about women’s rights, improving involvement in family and community decision-making and improving financial capacity. The aim of the youth associations is to encourage involvement in sub-district activities, to access skills training and to access income-generation projects.

**Fig 1:**
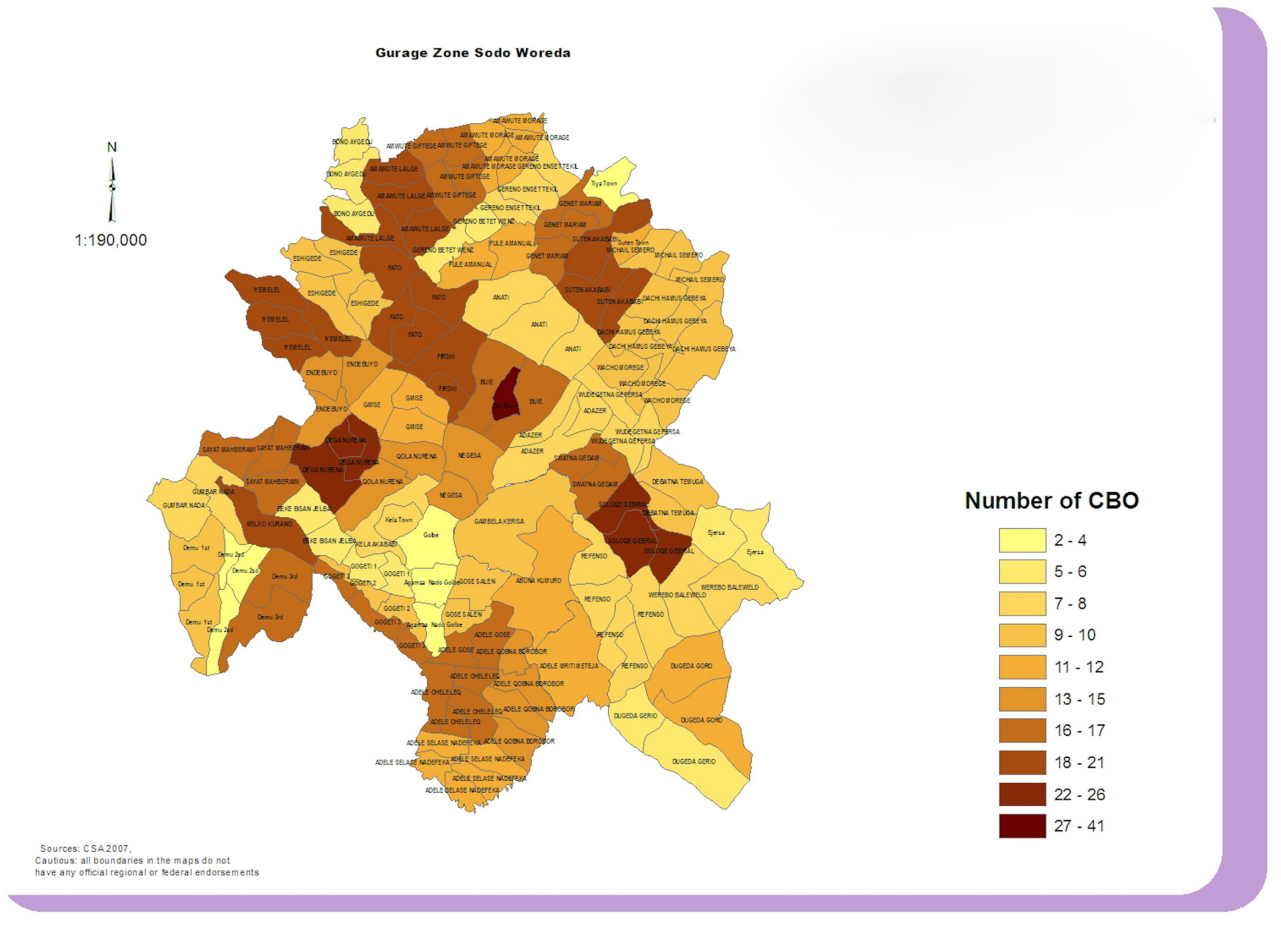
Distribution of Community-based Organisations (CBOs) in Sodo District, Gurage Zone Ethiopia.

Fig 1 indicates that CBOs are unevenly distributed across the district (range 2–41 per kebele). In general higher number of CBOs are found in the Northern and Western kebeles, which are in the highlands, and lower numbers in the lowland areas of the South and East. There is also a concentration in some urban areas e.g. Bui town.

There are two major NGOs in the district: Sodo-Bui Development Association (SBDA) and Compassion. The SDBA works in eight sub-districts. The primary aim is to support disadvantaged children through school and medical sponsorship and providing livelihood support for parents or caregivers. Compassion works in collaboration with protestant churches to provide medical and school expenses to vulnerable children in eight sub-districts. In addition they provide additional tutoring and life skills training to these children.

### Economic and religious resources

Farming is the major economic activity in the district. Small businesses and government employment are also other means of employment. Wheat, sorghum, maze, beans, *teff* and false banana (*enset*) are the most common harvest types in the district. There are 144 public sector agricultural extension workers who support farmers providing expertise in harvesting, natural resources and animal health.

There are 51 government microfinance associations across the district. The aim of the associations is to provide business skills training and offer loans to individuals and groups for income-generating activities.

There are on average two Orthodox Christian churches in each sub-district (125 in total). Eleven mosques and 28 protestant churches were also identified across the district. Each church and mosque has at least one priest or imam. These religious leaders usually have a high status and powerful influence within the sub-district.

### Government administration

There is a government administration structure in every sub-district, which includes a sub-district chairperson, administrator and finance officer. They are responsible for ensuring that government initiatives are implemented. To do this they work in collaboration with public sector health resources, e.g. HEWs and HDA, religious leaders and CBOs e.g. Youth and Women’s Associations.

### School, justice and recreational resources

Five sub-districts do not have a primary school. The remaining sub-districts have one or two primary schools. There are two secondary schools in the district. There are 45 adult literacy groups, part of a national government scheme, which provide free adult education to any illiterate adult who wishes to participate.

There is an established justice system. Each sub-district has a social court that consists of a committee of community members, and resolves minor conflicts. There are three police stations and one ‘first instance’ court (dealing with issues not resolved in the social court) in the district. There are one or two community policemen in each sub-district. Their role is to ensure the safety of community members.

The main recreational facilities identified were alcohol outlets (traditional bars). In each sub-district there are around five such bars, which sell locally made alcohol e.g. *tella*, *areqe*. In addition to their recreational function, community leaders considered these an environmental risk factor for mental illness.

The district hosts one of the eight historical sights registered in Ethiopia by UNESCO, the Tiya monuments or stelae (whc.unesco.org). These monuments are the remains of an ancient Ethiopian culture and have been interpreted to have a funerary significance. There are 36 monuments covered with symbols. These monuments are said to be among the most important of the nearly 160 archaeological sites discovered so far in the Sodo district. The Tiya monuments are a major tourist attraction in the area and source of income for some of the community members.

## Discussion

There is much potential for existing community resources to be used to positive effect to improve mental health services in low-income settings. It is therefore imperative to systematically identify resources. Resource mapping has previously been used to identify diverse assets with potential positive influences on health, for example child care educators [28], cancer screening facilities [31], community-based organisations [28,37] and green spaces which can be used for physical activity [19,28,32].

In addition to identifying strengths, resource mapping can also be used to recognize population level risk factors. For example, the high proportion of urban shops in which only “junk food” was available was identified in North American resource mapping studies [19,32]. Resource mapping has also been used to design community-based, sustainable interventions. It was used to design a family planning programme in Kenya [37] and a community initiative to reduce television viewing amongst children in New York [28]. There are few previous studies in low-income settings that have used resource mapping to develop community interventions [37], and none in mental health to our knowledge.

### Implications for mental health care plan

The results of this study indicate that beyond the available biomedical resources there are also substantial and varied community resources in a typical rural Ethiopian community. These community resources may be capitalised upon to enhance mental health care delivery and contribute to reducing the treatment gap for mental illness. There is an indication that community resources are unevenly distributed across the district, with CBOs more highly concentrated in the poorer highland areas compared to the wealthier lowland areas. This suggests that community resources are not necessarily associated with material wealth and may even have a more important function where material resources are fewer.

We propose that existing resources could potentially support the following aspects of the MHCP in Sodo district:

#### 1. Community awareness-raising and improving mental health literacy

The HEWs, HDA and leaders of community and religious organisations, for example *eddir* leaders and priests, could be involved in community awareness-raising activities. These individuals are known and respected in the community, meaning messages they convey are likely to be listened to. In addition mental health modules can be added to Community Conversations. The impact of community awareness-raising could be to reduce stigma, reduce human rights abuses and improve social integration of people with mental illness.

#### 2. Detection of persons with mental disorders

The HEWs and HDA can be trained to identify and refer persons with mental disorders such as psychosis and epilepsy, and those with severe depression and severe alcohol use problems to the health centre for treatment. These HEWs have access to all households within the sub-district so therefore are well placed to identify people with mental illness, including those who may be hidden from the wider community. They also have strong links to the health centre so can facilitate referral.

#### 3. Improving access to medical care

HEWs’ role in improving access to medical care can include:

(i) Reinforcing the message that continuing care is required even when the individual is symptom free, as part of their own routine house to house visits (ii) Reminding individuals to attend routine appointments at the health centre (iii) Asking about physical health and referring for review at the health centre or referring for review and (iv) Monitoring for relapse and referring for review at the health centre if required.

Community members, traditional healers, and community leaders also could help to facilitate people with mental illness to access medical care. This could be through (i) Informing affected individuals and their families that treatment is available and (ii) Assisting with practical aspects of transportation to the health centre, particularly for people with psychosis who are unwell and have difficult behaviour.

#### 4. Support with treatment adherence

Holy water priests and holy water attendants could receive education to encourage them to recommend that anti-psychotic or anti-epileptic medication is taken alongside using holy water. These individuals may have a strong influence over the behaviour of individuals with severe mental illness and their families. HEWs and the HDA could also support medication adherence by reminding individuals to take medication, and reinforcing the messages about the importance of adherence that will be given at the health centre.

#### 5. Rehabilitation and reactivation of social networks

Community-based rehabilitation, delivered by specially trained fieldworkers, will form one component of the MHCP. There are numerous targets for reintegration of people with mental illness into community activities and social networks. Community and religious leaders could be engaged by CBR fieldworkers to help facilitate the individuals’ participation. These community activities include (i) Religious activities such as attending church, mosque and religious groups such as *tsewa*. These religious institutions already have an active role with regard to the social and emotional support of community members. (ii) Participation in *eddir*, through financial or practical contributions (iii) Participation in women’s and youth associations and (iv) Participation in political activities, for example meetings of members of sub-districts.

In addition there are community resources that could be accessed to facilitate specific aspects of rehabilitation. These include microfinance groups for livelihood activities, and the adult literacy groups for education.

#### 6. Financial and practical support

*Eddir* groups may be willing to use collective funds to support individuals with severe mental illness and their families who are experiencing severe hardship. Practical support, for example help with rebuilding a house, could also be offered by these groups.

#### 7. Reducing physical restraint and physical abuse

The most important factor in reducing physical restraint will be access to medical treatment (See points 2–4). HEWs and the HDA may have a role in supporting the release from chains at home once an individual’s condition has improved. Targeted awareness-raising with holy water priests, delivered by HEWs, may help to reduce the practice of chaining and physical abuse.

#### Identification of environmental risk factors

A large number of alcohol establishments were found in the district, which implies high consumption rates and demand for alcohol. This finding strengthens the rationale for including alcohol use disorders as one of the priority areas for intervention in the MHCP. Identifying the alcohol establishments also provides a potential target for public health action to reduce alcohol consumption.

### Strengths and limitations

The participatory process of data collection is a strength of this study. The HEWs collected data in the community where they live and work, so they were able to supplement the information they gathered with self-knowledge. It is recognised that using local people to collect data can improve coverage and community acceptance of the research [19]. Data collected from community key informants was triangulated through discussions with district-level officials and community representatives, increasing the validity of the data.

Resource mapping allowed a holistic and comprehensive assessment of how the community level aspects of the MHCP could be operationalised. This will allow us to better meet the needs of individuals with mental illness, particularly with regard to recovery and reintegration. Resource mapping is one aspect of a strengths-based approach. This approach is in contrast to the needs-based approach to community development, which tends to perpetuate rather than eliminate needs due to its focus on deficiencies [29].

A limitation of this study is that a validated instrument was not used for data collection. Furthermore, we did not use Geographic Information Systems technology [19,31], which means we were not able to precisely locate the resources. A disadvantage of resource mapping is that it only gives us information about a given community’s resources, not how to use them. We can only speculate on the potential capacity of each resource to support mental health services. Additional development work may be needed in order to access some resources, and others may never prove useful or accessible in relation to the provision of mental healthcare. In particular, it is not well understood whether and how traditional healers can be involved in the ways we have suggested, for example encouraging anti-psychotic medication adherence. There may be a conflict of interest for traditional healers; there may be concern that encouraging use of medical treatments will reduce demand for their own services. These considerations call for interventions to enhance the potential roles of traditional and religious healers as part of the implementation of mental health care in the district. The sustainability of utilising existing resources, for example *eddir* groups, is also not known. There is evidence from a community mental health project in India that relying on community structures alone is not sustainable without external input or follow up [21].

## Conclusion

This study highlights the advantages of resource mapping for the development of a mental health care plan in a low-income setting. There are rich social and community resources available in Sodo district that will enhance the delivery of mental health care. These resources are likely to be available in most Ethiopian communities, so we anticipate these activities are scalable. Involving the community and its resources in mental health care is comprehensive, cost-effective and sustainable mechanism.

## Supporting Information

S1 File Community resource mapping tool (DOCX)
