# Supplementary material for: Beyond the Biomedical: Community Resources for Mental Health Care in Rural Ethiopia
Source: PLoS One. 2015 May 11;10(5):e0126666. doi: 10.1371/journal.pone.0126666 (PMC4427185; doi:10.1371/journal.pone.0126666)
Supplement: S1 File — (DOCX) [file pone.0126666.s001.docx]

**Supplementary information 1: Community Resource Mapping Tool**

| **Community Resource Mapping Tool for use in Rural Ethiopia (PRIME)**  **Note for the data collector: The purpose of this resource mapping is to provide a holistic and comprehensive picture of the community. Gathering and utilizing meaningful and accurate information about the community resources, infrastructures and needs is crucial to understand the resources that can be used by the community to bring about effective and lasting change. We aim to use the identified resources in the PRIME project to support the implementation of mental healthcare. However, beyond PRIME, the resources may be used to support the development of the Sodo district.** | | |
| --- | --- | --- |
|  | Date [ ] [ ]/ [ ] [ ]/[ ] [ ][ ] [ ] | |
|  | Supervisor ID [ ] [ ] | |
|  | Interviewer ID [ ] [ ] | |
| **1** | **General Information** | |
| 1.1 | Sub district (‘*Kebele’*) name | |
| 1.2 | Total population in the sub-district | [ ] [ ][ ] [ ][ ] |
| 1.3 | Total male population in the sub-district | [ ] [ ][ ] [ ][ ] |
| 1.4 | Total female population in the sub-district | [ ] [ ][ ] [ ][ ] |
| 1.5 | Total under 15 population in the sub-district | [ ] [ ][ ] [ ][ ] |
|  |  |  |
| **2** | **Schooling** |  |
| 2.1 | Total number of schools in the sub-district | [ ] [ ] |
| 2.2 | Primary schools |  |
| 2.2.1 | Total number of primary schools in the sub-district | [ ] [ ] |
| 2.2.2 | List of primary schools in the sub-district |  |
| 2.3 | Secondary schools |  |
| 2.3.1 | Total number secondary schools in the sub-district | [ ] [ ] |
| 2.3.2 | List of secondary schools in the sub-district |  |
| 2.4 | Technical and vocational schools |  |
| 2.4.1 | Total number of technical and vocational schools in the sub-district | [ ] [ ] |
| 2.4.2 | List of technical and vocational schools in the sub-district |  |
| **3** | **Religious Institutions** |  |
| 3.1 | Total number of religious institutes in the sub-district | [ ] [ ] |
| 3.2 | Orthodox Churches |  |
| 3.2.1 | Total number orthodox churches in the sub-district | [ ] [ ] |
| 3.2.2 | List of orthodox churches |  |
| 3.3 | Mosques |  |
| 3.3.1 | Total number of mosques | [ ] [ ] |
| 3.3.2 | List of mosques |  |
| 3.4 | Other religious institutions |  |
| 3.4.1 | Total number of other religious institutions | [ ] [ ] |
| 3.4.2 | List of other religious institutes |  |
| **4** | **Justice** |  |
| 4.1 | Police stations |  |
| 4.1.1 | Total number of police stations | [ ] [ ] |
| 4.1.2 | List of police stations |  |
| 4.2 | Social courts |  |
| 4.2.1 | Total number of social courts | [ ] [ ] |
| 4.2.2 | List of social courts |  |
| **5** | **Recreation Facilities** |  |
| 5.1 | Hotels |  |
| 5.1.1 | Total number of hotels | [ ] [ ] |
| 5.1.2 | List of hotels |  |
| 5.2 | Cafés |  |
| 5.2.1 | Total number of cafes | [ ] [ ] |
| 5.2.2 | List of cafe |  |
| 5.3 | *‘Telabet’* (Home brewed beer vendor) |  |
| 5.3.1 | Total number of ‘*telabets’* | [ ] [ ] |
| 5.3.2 | List of *‘telabets’* |  |
| 5.4 | *‘Tejbet’* (Home brewed honey wine vendor) |  |
| 5.4.1 | Total number of ‘*tejbets*’ | [ ] [ ] |
| 5.4.2 | List of*‘tejbets’* |  |
| 5.5 | *‘Arekebet’* (Home distilled spirit vendor) |  |
| 5.5.1 | Total number of ‘*arekebets*’ | [ ] [ ] |
| 5.5.2 | List of *‘arekebet’* |  |
| 5.6 | Other recreational facilites |  |
| 5.6.1 | Total number of other recreational facilities | [ ] [ ] |
| 5.6.2 | List of other recreational facilities |  |
| **6** | **Civil Society Organizations (CSOs)** |  |
| 6.1 | Total number CSOs | [ ] [ ] |
| 6.2 | List of civil society organisations |  |
| 6.3 | Community based organizations (CBOs) |  |
| 6.3.1 | Total number of CBOs | [ ] [ ] |
| 6.3.2 | List of CBOs |  |
| 6.4 | Faith Based Organizations (FBOs) |  |
| 6.4.1 | Total number of FBOs | [ ] [ ] |
| 6.4.2 | List of FBOs |  |
| 6.5 | Non Governmental Organizations (NGOs) |  |
| 6.5.1 | Total number of NGOs | [ ] [ ] |
| 6.5.2 | List of NGOs |  |
| **7** | **Community associations** |  |
| 7.1 | Total number of community associations in the sub-district | [ ] [ ] |
| 7.2 | List of community associations |  |
| 7.3 | Women’s fora |  |
| 7.3.1 | Total number of women’s fora | [ ] [ ] |
| 7.3.2 | List of women’s fora |  |
| 7.4 | Youth forums |  |
| 7.4.1 | Total number of youth fora | [ ] [ ] |
| 7.4.2 | List of youth fora |  |
| 7.5 | Resident’s forums |  |
| 7.5.1 | Total number of resident’s fora | [ ] [ ] |
| 7.5.2 | List of resident’s fora |  |
| 7.6 | *Eddirs* |  |
| 7.6.1 | Total number of *eddirs* | [ ] [ ] |
| 7.6.2 | List of *eddirs* |  |
| 7.7 | *Mahabers* |  |
| 7.7.1 | Total number of *‘mahabers’* | [ ] [ ] |
| 7.7.2 | List of *‘mahabers’* |  |
| 7.8 | 1 in 5 groups |  |
| 7.8.1 | Total number of 1 in 5 groups | [ ] [ ] |
| 7.9 | Health Development Army (HDA) |  |
| 7.9.1 | Total number of HDA | [ ] [ ] |
| 7.10 | Agricultural development agents (ADA) |  |
| 7.10.1 | Total number of ADA | [ ] [ ] |
| 7.10.2 | Total number of ADA with animal health specialisation | [ ] [ ] |
| 7.10.3 | Total number of ADA with crop production specialisation | [ ] [ ] |
| 7.10.4 | Total number of ADA with forest protection specialisation | [ ] [ ] |
| 7.11 | Adult literacy program |  |
| 7.11.1 | Total number of adult literacy program | [ ] [ ] |
| **8** | **Other Community Assets** |  |
| 8.1 | Traditional Healers |  |
| 8.1.1 | Total number of traditional healers | [ ] [ ] |
| 8.2 | Herbalist |  |
| 8.2.1 | Total number of herbalist | [ ] [ ] |
| 8.2.2 | List of herbalists |  |
| 8.3 | *Tenquay* (‘witch doctor’) |  |
| 8.3.1 | Total number of *tenquay* (‘witch doctor’) | [ ] [ ] |
| 8.3.2 | List of *tenquay* (‘witch doctor’) |  |
| 8.4 | *Wogesha* (Bone setters/ traditional physiotherapists) |  |
| 8.4.1 | Total number of *wogesha* | [ ] [ ] |
| 8.4.2 | List of *wogesha* |  |
| 8.5 | Traditional Birth Attendants (TBAs) |  |
| 8.5.1 | Total number of TBAs | [ ] [ ] |
| 8.5.2 | List if TBAs |  |
| 8.6 | Microfinance institute |  |
| 8.6.1 | Total number of microfinance institute | [ ] [ ] |
| 8.7 | Physical and Land assets |  |
| 8.7.1 | Agriculture type, trees/ forest |  |
| 8.7.2 | List of common agricultural /harvest type |  |
| 8.9 | Lake, ponds, streams |  |
| 8.9.1 | List of lake, ponds, streams |  |
| 8.10 | Parks |  |
| 8.10.1 | List of parks |  |
| 8.11 | Animals (endemic) |  |
| 8.11.1 | List of endemic animals |  |
| 8.12 | Holy water (*tsebel*) sites |  |
| 8.12.1 | Total number of *tsebel* sites | [ ] [ ] |
| 8.12.2 | List of *tsebel* sites |  |
| 8.13 | Historical Sites |  |
| 8.13.1 | Total number of historical sites | [ ] [ ] |
| 8.13.2 | List of historical sites |  |

Name of data collectors

1._______________________________2. _______________________________

Data source (key informant/s and other sources of information)

_____________________________________________________________________

_____________________________________________________________________

_____________________________________________________________________

Name of supervisor __________________Signature___________

Date______________
